# Lactate Induces Pro-tumor Reprogramming in Intratumoral Plasmacytoid Dendritic Cells

**DOI:** 10.3389/fimmu.2019.01878

**Published:** 2019-08-07

**Authors:** Deblina Raychaudhuri, Roopkatha Bhattacharya, Bishnu Prasad Sinha, Chinky Shiu Chen Liu, Amrit Raj Ghosh, Oindrila Rahaman, Purbita Bandopadhyay, Jafar Sarif, Ranit D'Rozario, Santu Paul, Anirban Das, Diptendra K. Sarkar, Samit Chattopadhyay, Dipyaman Ganguly

**Affiliations:** ^1^IICB-Translational Research Unit of Excellence, Division of Cancer Biology and Inflammatory Disorders, CSIR-Indian Institute of Chemical Biology, Kolkata, India; ^2^Academy of Scientific and Innovative Research, Kolkata, India; ^3^Mass Spectrometry Core Facility, IICB-Translational Research Unit of Excellence, CSIR-Indian Institute of Chemical Biology, Kolkata, India; ^4^Department of General Surgery, Institute of Postgraduate Medical Education and Research, Kolkata, India

**Keywords:** breast cancer, FoxP3^+^ T cells, lactate, plasmacytoid dendritic cells, type I IFN

## Abstract

Plasmacytoid dendritic cells are the most efficient producers of type I interferons, viz. IFNα, in the body and thus have the ability to influence anti-tumor immune responses. But repression of effective intra-tumoral pDC activation is a key immuno-evasion strategy exhibited in tumors—tumor-recruited pDCs are rendered “tolerogenic,” characterized by deficiency in IFNα induction and ability to expand regulatory T cells *in situ*. But the tumor-derived factors that drive this functional reprogramming of intra-tumoral pDCs are not established. In this study we aimed at exploring if intra-tumoral abundance of the oncometabolite lactate influences intra-tumoral pDC function. We found that lactate attenuates IFNα induction by pDCs mediated by intracellular Ca^2+^ mobilization triggered by cell surface GPR81 receptor as well as directly by cytosolic import of lactate in pDCs through the cell surface monocarboxylate transporters, affecting cellular metabolism needed for effective pDC activation. We also found that lactate enhances tryptophan metabolism and kynurenine production by pDCs which contribute to induction of FoxP3^+^ CD4^+^ regulatory T cells, the major immunosuppressive immune cell subset in tumor microenvironment. We validated these mechanisms of lactate-driven pDC reprogramming by looking into tumor recruited pDCs isolated from patients with breast cancers as well as in a preclinical model of breast cancer in mice. Thus, we discovered a hitherto unknown link between intra-tumoral abundance of an oncometabolite resulting from metabolic adaptation in cancer cells and the pro-tumor tolerogenic function of tumor-recruited pDCs, revealing new therapeutic targets for potentiating anti-cancer immune responses.

## Introduction

Cancer immunosurveillance mechanisms recognize transformed cells in the body to prevent neoplastic growth (1). Importance of type II interferon (IFN) in mediating efficient anti-tumor immune response is well-established (2, 3). But in addition, an essential role of type I IFNs has also been described in driving tumor rejection via both cancer cell intrinsic and extrinsic effects (3–8). Given the importance of type I IFNs in anti-tumor immune response, it is imperative for cancer cells to adopt strategies to evade either induction or function of these cytokines in the tumor bed.

Plasmacytoid dendritic cells (pDCs) are the major producers of type I IFNs (9). Activation of endosomal toll-like receptors (TLRs), in human pDCs, in response to foreign nucleic acids is crucial for anti-viral immunity (10). A critical role of pDC-derived type I IFNs is also established in different clinical contexts of autoreactive inflammation (11–18). Although type I IFNs play a critical role in anti-tumor immune response, previous studies have reported pDC dysfunction and acquisition of tolerogenic function in the tumor bed (19–21), thus providing evidence that cancer cells do adopt immunoregulatory strategies to evade intra-tumoral activation of pDCs. Tumor-recruited pDCs have been shown to lack IFNα induction and drive expansion of regulatory T cells (Tregs) in different cancers (19, 20). Tregs (characterized by the master regulator transcription factor FoxP3) prevent aberrant activation of the immune system against self-antigens thus preventing autoimmunity (22). But, presence of Tregs in tumor bed is associated with poor prognosis in various types of cancers (23–25). The immune-regulatory mechanisms operative in the tumor bed that inhibit induction of type I IFNs by recruited pDCs and augment their ability to induce Tregs remain elusive.

To support the proliferative phenotype, cancer cells adapt metabolic changes such as the Warburg effect (26). The major oncometabolite resulting from this glycolytic switch is lactate, which is abundant in tumor microenvironment (26, 27). Interestingly, an immunoregulatory role of lactate abundance is evident from its effect on expansion of myeloid derived suppressor cells, inhibition of NK cell and T cell mediated cytotoxicity, anti-inflammatory M2 polarization of tumor associated macrophages in tumor microenvironment as well as suppression of pro-inflammatory cytokine production by dendritic cells (28–31). In the present study we explored if this major oncometabolite also regulated intra-tumor pDC function in breast cancer. We found that cancer cell-derived lactate attenuates activation of human pDCs in response to TLR9 ligand and consequent type I IFN induction. On mechanistic exploration we attributed this to intracellular Ca^2+^ mobilization driven by cell surface lactate receptor GPR81, the lactate receptor on pDC surface, as well as cytosolic import of lactate itself via monocarboxylate transporters. We also explored the impact of lactate on the ability of pDCs to induce Tregs and implicated lactate-induced modulation of tryptophan metabolism in pDCs in the expansion of CD4^+^ FoxP3^+^ Tregs in breast cancer. These findings were validated by looking at tumor-recruited pDCs from breast cancer patients as well as in a murine syngeneic model of breast cancer. Thus, we discovered a link between a major metabolic adaptation of cancer cells and a critical immune-evasion mechanism driven by them by modulating the functional phenotype of tumor-recruited pDCs.

## Materials and Methods

### Plasmacytoid Dendritic Cell Culture

PBMCs were isolated using density gradient centrifugation (Histopaque, Ficol) from blood drawn from healthy donors (after obtaining informed consent and approval by the Institutional Ethics Committee and in accordance with the Declaration of Helsinki). For some experiments, human pDCs were also isolated from buffy coats collected from Tata Medical Centre Blood Bank, Kolkata, through an approved material transfer agreement, in concurrence with the institutional human ethics committee. PDCs were sorted from whole PBMCs by magnetic immunoselection, using anti-BDCA4 microbeads (Miltenyi Biotec, Germany) and cultured in 100 μl of complete RPMI media (GIBCO), at 37°C and 5% CO_2._ pDCs in culture were treated as mentioned in the figure legends. CpG-ODN (Invivogen, USA), Potassium lactate, EGTA, Cyclosporin A (Sigma-Aldrich, St. Louis, MO, USA), Gallein, 8-Bromo cAMP sodium salt, AR-C155858 (Tocris Biosciences, Bristol, UK), and CAMKII Inhibitor (Calbiochem, USA) were used for treating pDCs as indicated.

### Enzyme-Linked Immunosorbent Assay

Concentration of IFNα in pDC culture supernatants was determined using sandwich ELISA (Mabtech, Sweden) according to manufacturer's protocol. Concentration of IFNα in peritoneal fluid of BALB/c mice was determined by sandwich ELISA using 1:1,000 dilution of RMMA-1 primary antibody (pbl interferonsource,USA), 1:500 dilution of polyclonal rabbit antibody to Mouse IFN Alpha (pbl interferonsource) and 1:10,000 dilution of anti-rabbit IgG, HRP conjugated tertiary antibody (Cell Signaling, USA).

### Gene Knockdown Experiments

Freshly isolated pDCs were allowed to recover in complete media in the incubator for 1 h followed by a PBS wash. Then cells were resuspended in 100 μl of supplemented P3 buffer (Amaxa Lonza 4D nucleofector kit, Koln, Germany) and either control siRNA (esiRNA targeting EGFP, Sigma-Aldrich) or GPR81 target siRNA (sequence: GUUGCAUCAGUGUGGCAAAdTdT, Eurogentec, Belgium) was added following which cells were nucleofected using the preset FF168 protocol in an Amaxa Lonza 4D nucleofector. Nucleofected cells were kept in culture for 16 h following which they were collected, counted, plated and treated as indicated.

### Calcium Mobilization Assay

Isolated primary pDCs were stained with calcium binding dye, Fluo 3-AM (1.5 μM), for 30 min in PBS containing 1.2 mM CaCl_2_ and 2% FBS (GIBCO) at 37°C. Following incubation, the cells were washed twice in the same buffer and allowed to rest for 30 min at room temperature to allow efflux of excess dye. Stained cells were then acquired on a BD Fortessa flow cytometer for indicated time periods before and after the addition of specified treatments. The change in MFI in the FITC channel (Fluo 3-AM) was indicative of the difference in intracellular calcium mobilization upon addition of treatment.

### RNA Isolation and Real Time PCR

Total RNA was isolated from nucleofected pDCs, from pDCs isolated from tumor tissue, blood as well as mouse peritoneal cells using the TriZol method according to the manufacturer's protocol and reverse transcribed to form cDNA (using Superscript III cDNA kit from Invitrogen, USA). The cDNA was used for various gene expression studies via Real Time PCR (Applied Biosystems 7500 Fast, USA). Primers used for real time PCR are listed in Supplementary Table S1.

### Measurement of Extracellular Acidification Rate (ECAR)

Glycolysis Stress Test was done to measure the ECAR values of non-transfected, EGFP siRNA transfected (control), and GPR81 siRNA transfected pDCs seeded on poly-L-Lysine (Sigma-Aldrich) coated wells of a 24 well-plate, and pre-treated with the reagents indicated in the figure legend for 4 h before being subjected to XF-24 Analyzer (Seahorse Biosciences). The experiments were carried out according to the Manufacturer's protocol, using the reagents (10 mM D-glucose, 1 mM oligomycin, 100 nM 2-Deoxy D-glucose) and XF media (supplemented with 4 mM L-glutamine) supplied by the manufacturer.

### Patient Samples

Patients with breast cancer were recruited from Department of General Surgery, Institute of Postgraduate Medical Education & Research (IPGMER), Kolkata, India, as per recommendations of the Institutional Review Boards of IPGMER as well as CSIR-IICB. All recruited patients had invasive ductal carcinoma of the breast without any organ metastasis (Supplementary Table S2). A portion of tumor tissue samples resected during Modified Radical Mastectomy, were collected after obtaining informed consent from the donors as well as ethical clearance by the concerned institutions.

### Sorting of pDCs From Human Breast Tumor Tissue

Collected tumor tissue was washed twice with PBS to remove contamination from exogenous blood, before being minced into tiny pieces and digested for 2 h at 37°C under shaking condition in a digestion buffer containing 1 mg/ml type I collagenase (Himedia) and 0.15 g/ml BSA (Himedia). Following digestion the mixture was centrifuged at 500 rpm for 5 min and the supernatant was collected and passed through a 70 μ strainer. Then the supernatant was spun at 1,500 rpm for 5 min to obtain the cell pellet which was stained with CD45 FITC, CD3 PerCP, CD19 APC, BDCA4 PE, CD123 BV421 (BD Biosciences), and pDCs were sorted in a moFLO cell sorter. The isolated pDCs were either subjected to gene expression studies or co-cultured with naïve CD4^+^ T cells.

### Naive CD4^+^ T Cell Isolation and Co-culture With pDCs

CD4^+^ T cells were isolated from PBMCs by magnetic immunoselection using anti-CD4 microbeads (Miltenyi Biotec, Germany). Similarly, naive T cells were sorted from the isolated CD4^+^ T cells using anti-CD45RA microbeads before being co-cultured with autologous pDCs in a 5:1 ratio for 5 days in supplemented RPMI media.

### Flow Cytometric Analysis of FoxP3^+^ CD4^+^ T Cells

T cells along with untreated or lactate treated pDCs from 5 day old co-cultures were either stained with BDCA2 APC or CD4 BUV395 to differentiate the T cells from pDCs during flow cytometry. This was followed either only by intracellular staining with FoxP3 PE (clone: 259D/C7, BD Biosciences) or in some cases by surface staining with CD25 APC followed by intracellular staining with FoxP3 PE (clone: PCH101, eBioscience) and acquisition in a flow cytometer.

### Suppression of T Cell Proliferation Assay

Flow cytometry assisted cell sorting in a moFLO cell sorter was used to isolate CD4^+^CD25^high^CD127^low^ cells from pDC T cell co-cultures. CD25 efluor450, CD127 APC-Cy7 and CD4 FITC (BD Biosciences) were used for staining. These were in turn co-cultured for 5 days with autologous Cell Trace Violet (Invitrogen) stained CD45RA^+^CD4^+^ T cells stimulated with CD3/CD28 (5 μg/ml) and subsequently subjected to flow cytometry to determine the degree of cell proliferation from the extent of CTV dilution.

### L-Kynurenine and Tryptophan Quantitation by Mass Spectrometry

Cell culture supernatants were extracted in a 1:4 ratio with acetonitrile +0.1% formic acid (J.T. Baker), by intermittent vortexing and incubation on ice followed by centrifugation at 14,000 rpm for 10 min at 4°C. Then the supernatant was subjected to LC-MS/MS for L-kynurenine and tryptophan quantitation. Standard solutions of L-kynurenine (Sigma Aldrich) and tryptophan (Sigma) were prepared in the same acetonitrile- formic acid mixture. Standards having concentrations from 2.5 to 62.5 ng/ml were prepared for tryptophan and 2.5–125 ng/ml for were used for kynurenine. LTQ ORBITRAP XL and Hypersil Gold C18 column with a diameter of 100 × 2.1 mm, particle size 1.9 μ, was used for the mass spectrometry. The column (stationary phase) was maintained at 40°C. A mixture of Solution A (H_2_O +0.1% formic acid) and solution B (acetonitrile + 0.1% formic acid) formed the mobile phase. Injection volume was 10 μl and the samples were run in an isocratic system (40% acetonitrile). The cut off for detection of intact L-kynurenine was (m/z) 209.09 and tryptophan was (m/z) 205.09. The retention time for L-kynurenine was 0.83 min and retention time for tryptophan was 0.87 min. The (m/z) for detection of fragmented kynurenine was 192.06 and for tryptophan was 188.07. Thermo Xcalibur software was used for analysis.

### *In vivo* Efficacy Assay

Female BALB/c mice, 6–8 weeks old, were divided into 3 groups (1-Vehicle, 2-CpGA/CpGB and 3-CpGA/CpGB+0.5 g/kg sodium lactate), each having 3 mice. The 6 mice in groups 2 and 3 were intraperitoneally injected with 25 μg of CpGA+ 25 μg of CpGB, whereas the mice in the first group were i.p. administered with 1X PBS only. At indicated time points following CpG injection, the mice in the third group were i.p. injected with 0.5 g/kg lactate. After 14 h, the mice were sacrificed, peritoneal fluid wash collected and centrifuged to separate the liquid and the cellular components. The liquid component was subjected to mouse IFNα ELISA and cellular component was subjected to both flow cytometric analysis (to measure pDC infiltration) and gene expression studies (to assay expression of ISGs). All animal experiments were done on approval of the Institutional Animal Ethics Committee of CSIR-IICB.

### 4T1 Tumor Mouse Models

Six to eight weeks old female BALB/c mice were used for generating the syngeneic tumor model with 4T1 cells. All animal experiments were done on approval of the Institutional Animal Ethics Committee of CSIR-IICB. The mice were injected subcutaneously in the right flank with 1.5 × 10^6^ cells of the mouse breast cancer cell line 4T1. Once the tumors became visually apparent, the diameters of the tumors were measured at 3 different axes daily till the mice were sacrificed. When the tumors crossed an average diameter of 6.5 mm, the mice were assigned to 4 groups for daily intra-tumoral injections of PBS, gallein, ARC, or gallein+ARC. On the 5th day, the mice were sacrificed and tumors excised. The harvested tumor was partly collected in RNA Later (Qiagen) for subsequent RNA isolation and gene expression studies and washed, digested and stained for flow cytometry using the same protocol as described for human tumor tissue processing.

### Statistics

Paired Student's *t*-test, unpaired *t*-test, Wilcoxon matched paired *t*-test, Mann-Whitney *t*-test or Spearman's correlation test was done, as indicated in the figure legends using the GraphPad Prism 5.0 software.

## Results

### Lactate Inhibits Type I IFN Induction in pDCs

**Figure 1:**
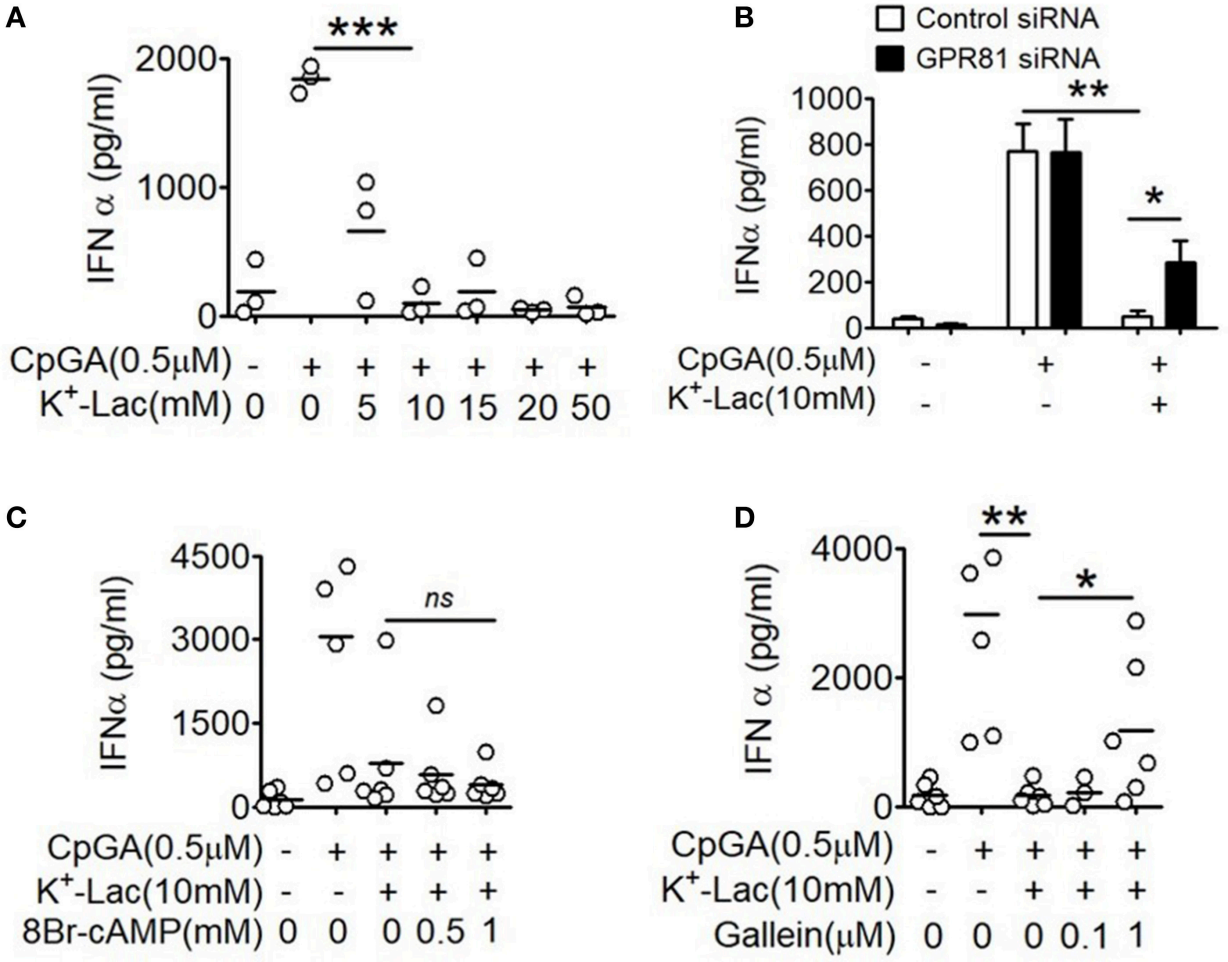
Effect of GPR81 on lactate-induced inhibition of type I IFN induction in pDCs. **(A)** Indicated concentrations of K^+^-lactate were added to overnight pDC cultures following which, IFNα concentration in the culture supernatants was estimated by ELISA. *n* = 3 from 2 independent experiments and two-tailed paired Student's *t-*test was done. **(B)** pDCs nucleofected with control siRNA or GPR81 siRNA, were treated with K^+^-lactate and stimulated with CpGA. After 20 h, ELISA was done to measure IFNα concentration in the culture supernatants. *n* = 6 from 3 independent experiments and one-tailed paired Student's *t-*test was done. Data is represented as Mean ± SEM. **(C,D)** K^+^-lactate was added to pDC cultures containing indicated concentrations of 8-Br cAMP **(C)** or gallein **(D)** and stimulated with CpGA. After 20 h of incubation IFNα concentration was measured in the culture supernatants. *n* = 5 from 2 to 3 independent experiments and one-tailed paired Student's *t*-test was done (^*^p < 0.05, ^**^p < 0.005, ^***^p < 0.0005, and ns, not significant).

In order to study the effect of lactate on type I IFN induction in pDCs, potassium lactate (K^+^-Lac) solution was added directly to the pDC cultures, revealing dose-dependent inhibition of type I IFN induction with significant reduction in IFNα at a concentration of 10 mM (Figure 1A). The extent of inhibition ranged from 40% to 100% in presence of 10 mM K^+^-lactate (Supplementary Figure S1A). We used 10 mM K^+^-Lac for further experiments, as this concentration of lactate did not affect the pH of the media significantly (Supplementary Figure S1B).

### Role of GPR81 in Lactate-Driven pDC Dysfunction

Extracellular lactate can communicate with cells through either the cell surface G-protein coupled receptor 81 (GPR81), or via direct import into the cells through lactate transporters on the cell surface, the monocarboxylate transporters (MCT)-1 and MCT-2 (32). Moreover, GPR81 has been shown to regulate the production of both pro as well as anti-inflammatory cytokines by intestinal antigen presenting cells in mice (33). Hence, to explore the role of GPR81 in mediating the effect of lactate on human pDCs, first we knocked down GPR81 in primary human pDCs by RNA interference (Supplementary Figure S2). We found that GPR81-deficient pDCs showed partial but significant reversal of the inhibition of IFNα induction in presence of lactate (Figure 1B). GPR81 is a G_i_ protein coupled receptor thus presumably driving typical G_i_ signaling downstream (34), involving reduction in cAMP generation driven by the Gα_i_ subunit and cytosolic Ca^2+^ mobilization driven by the Gβγ subunit. Addition of 8-bromo cAMP, a cell permeable cAMP analog, could not reverse the lactate-driven inhibition of pDC activation (Figure 1C), thus excluding the contribution from Gα_i_ subunit-mediated cAMP depletion.

In order to assess the effect of the Gβγ subunit signaling we used gallein, an inhibitor for Gβγ subunit. To optimize the inhibitory concentration of gallein, we tested the efficacy of a range of doses of gallein in preventing GPCR-mediated calcium influx in response to lactate as well as chemerin, the pDC-specific chemokine, used as a positive control since it interacts with its receptor CMKLR1 on pDCs, which is also a GPCR with Gi signaling and reported to cause calcium influx (35). We found a dose dependent decrease in calcium mobilization, driven by both chemerin and lactate, which were completely abrogated at 1 μM gallein concentration (Supplementary Figure S3). In presence of gallein at 1 μM concentration, there was again a significant reversal of the inhibitory effect of lactate on pDC activation (Figure 1D).

### GPR81 Activation Induced Ca^2+^ Mobilization Mediates Lactate-Induced pDC Dysfunction

**Figure 2:**
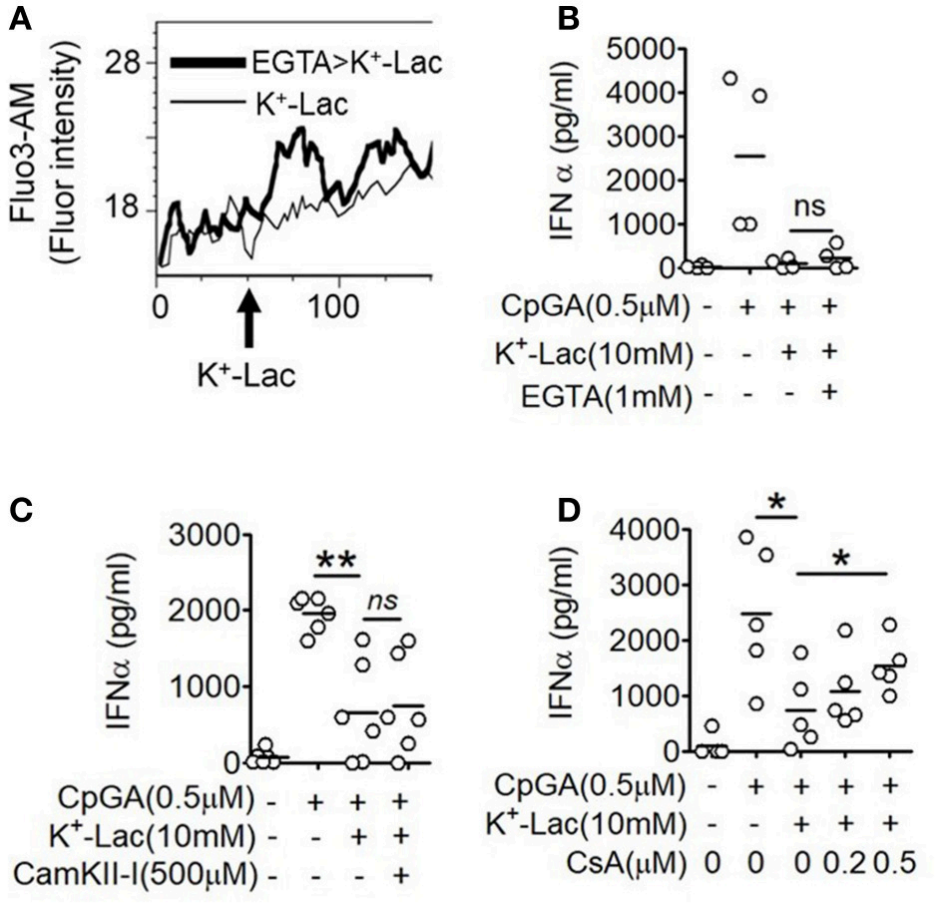
Role of intracellular Ca^2+^ mobilization in lactate-induced GPR81 signaling in pDCs. **(A)** Primary pDCs were stained with calcium binding dye- Fluo-3AM and in the presence (thick line) or absence (thin line) of 1 mM EGTA acquired on a flow cytometer, before and after addition of K^+^-lactate. The arrow represents the addition of 10 mM K^+^-lactate and the Y-axis represents the fluorescence emitted by the dye. The figures are representative of 3 independent experiments. **(B,C)** K^+^-lactate was added to pDC cultures preincubated with indicated concentrations of EGTA **(B)**, CamKII-I **(C)**, and CsA **(D)**, and then stimulated with CpGA. After 20 h, IFNα ELISA was done with the culture supernatants. *n* = 4–6 from 2 to 3 independent experiments and two-tailed paired Student's *t*-test (^*^p < 0.05, ^**^p < 0.005 and ns, not significant).

The major outcome of Gβγ signaling is cytosolic mobilization of Ca^2+^. On addition of lactate, pDCs showed instantaneous induction of cytosolic Ca^2+^ mobilization in a flow cytometry-based assay (Figure 2A). Also, in the presence of EGTA, the cell non-permeable Ca^2+^ chelator, the cytosolic free Ca^2+^ accumulation was not affected (Figure 2A) and inhibition of type I IFN induction by lactate could not be reversed (Figure 2B). These data indicated that lactate induces mobilization of Ca^2+^ from intracellular sources in pDCs rather than inducing influx of extracellular Ca^2+^. Intracellular Ca^2+^ mobilization is known to regulate downstream gene expression by either or both of Ca^2+^/calmodulin dependent protein kinase II (CAMKII), and calcineurin phosphatase (CALN) (36). We found that when CALN was inhibited, but not CAMKII, a significant reversal of the inhibitory effect of lactate was registered (Figures 2C,D). Thus, increased free cytosolic Ca^2+^, resulting from lactate-GPR81 interaction, engages CALN signaling, and this has a partial but significant contribution to the inhibitory effect of lactate on pDC activation and type I IFN induction.

### Role of Cytosolic Import of Lactate in pDC Dysfunction

**Figure 3:**
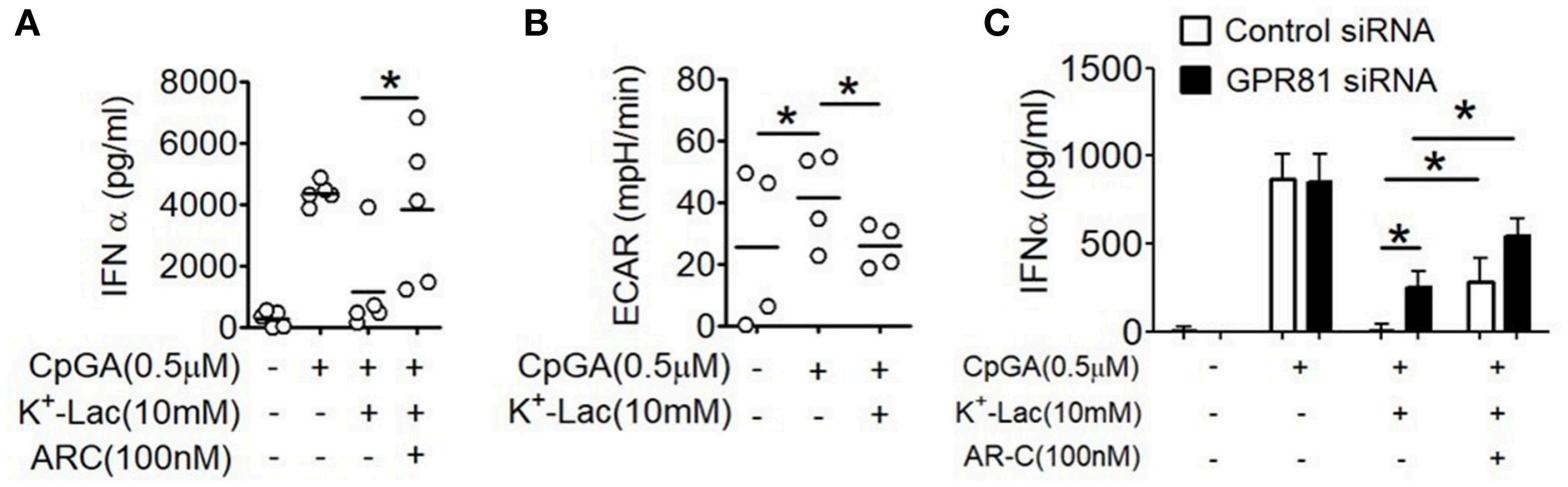
Effect of MCTs on lactate-induced inhibition of type I IFN induction in pDCs. **(A)** K^+^-lactate was added to pDC cultures preincubated with indicated concentrations of ARC, and then stimulated with CpGA. After 20 h, IFNα ELISA was done with the culture supernatants. *n* = 5 from 3 independent experiments and one-tailed Paired Student's *t*-test was done. **(B)** PDCs were subjected to the glycolysis stress test and ECAR values noted after the completion of the run. One-tailed Paired Student's *t*-test was done on the average ECAR values recorded over the three time points after oligomycin treatment. **(C)** pDCs nucleofected with control siRNA or GPR81 siRNA were incubated with ARC before addition of K^+^-lactate and CpGA. Following 20 h of culture, supernatants were assayed for IFNα by ELISA. *n* = 7 from 3 independent experiments and one-tailed paired Student's *t*-test was done. Data is represented as Mean ± SEM (^*^p < 0.05).

As discussed earlier, extracellular lactate can also influence pDCs through intracellular import via the monocarboxylate transporters. Primary human pDCs show significantly higher expression of MCT1 (Supplementary Figure S4). In order to explore this possibility, we pretreated pDCs with AR-C155858 (ARC), an inhibitor of MCT-1 and MCT-2 (37). Interestingly, ARC also resulted in significant reversal of the lactate-mediated inhibition of IFNα (Figure 3A). In a recent study it was shown that a glycolytic switch in cellular respiration was characteristic and essential for type I IFN induction by pDCs in response to TLR stimulation (38). A negative feed-back effect of cytosolic import of extracellular lactate via MCTs on activation-induced glycolytic switch is also reported in immune cells, viz. in human monocytes (39). We looked into the effect of lactate on this TLR9-induced glycolytic switch in human pDCs, by measuring extracellular acidification on a metabolic flux analyzer. We found that presence of extracellular lactate indeed hinders the TLR9-induced glycolytic switch, with significant reduction in extracellular acidification rate (ECAR) (Figure 3B). The MCT transporters significantly add to the contribution from GPR81 triggering in lactate-mediated inhibition of type I IFN induction, as there was significant enhancement of type I IFN induction in GPR81-deficient pDCs when MCTs were also inhibited concomitantly (Figure 3C). Thus, the inhibitory effect of lactate on TLR-activation in pDCs is mediated by non-redundant contributions from cell surface GPR81 triggering as well as cytosolic import via the MCT transporters.

We found that treating healthy human pDCs with lactate in culture upregulates expression of both GPR81 and MCT1 genes (Supplementary Figures S5A,B). To validate if this is also true in the lactate-rich tumor microenvironment in human patients, we looked at breast cancer, as intra-tumoral pDC-dysfunction is well-documented in these patients (19, 20). We collected tumor tissues from breast cancer patients, isolated intratumoral pDCs as well as pDCs from peripheral blood of the same patients by flow cytometry assisted cell sorting and performed gene expression studies on them. We found intratumoral pDCs from these patients as well have enhanced expression of both GPR81 (Supplementary Figure S5C) and MCT1 (Supplementary Figure S5D) compared to their peripheral counterparts.

### Lactate Enhances Regulatory T Cell Induction by pDCs

**Figure 4:**
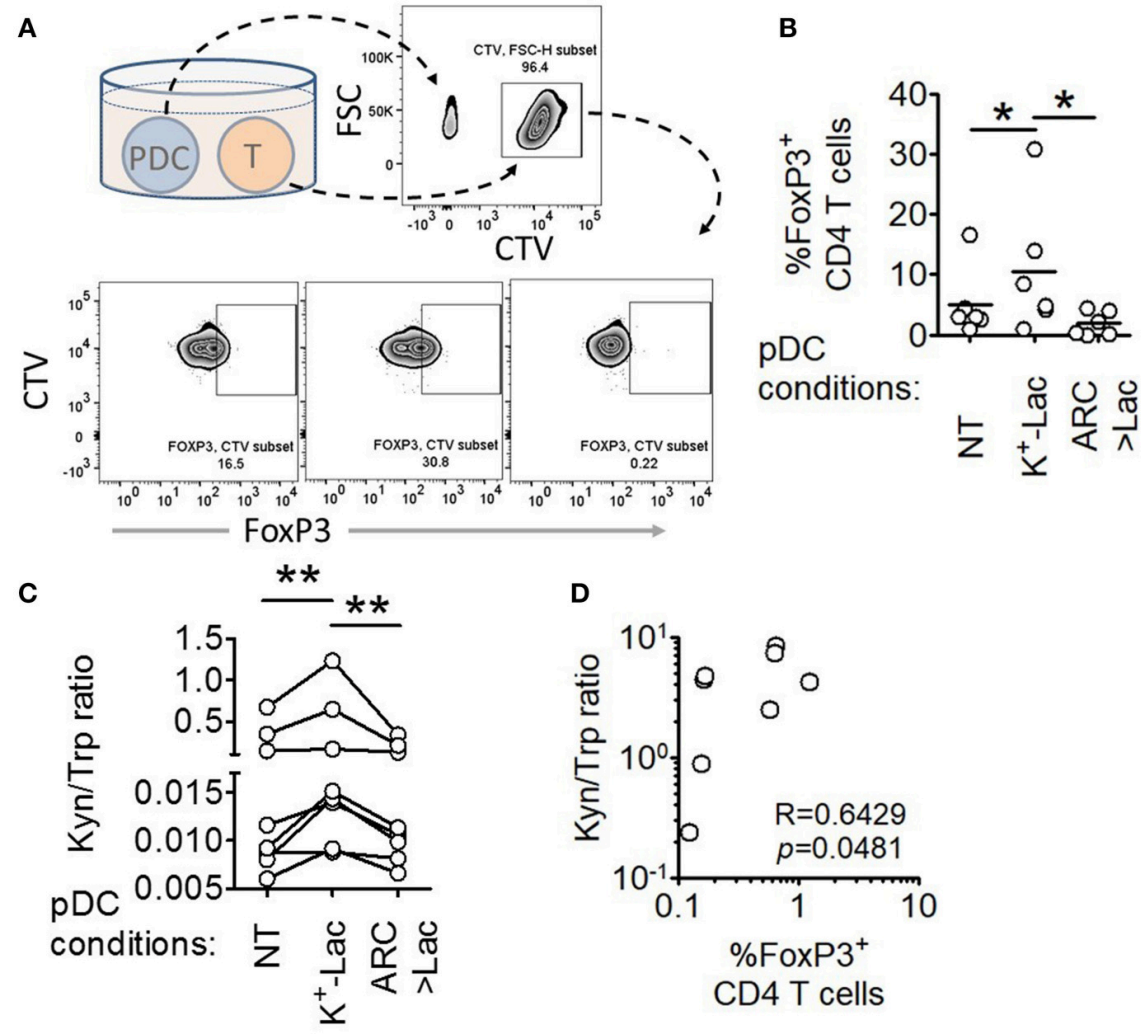
Lactate drives human pDCs to generate FoxP3^+^ T regulatory cells. **(A,B)** Autologous CD45RA^+^CD4^+^ T cells were co-cultured with untreated pDCs, K^+^-Lac treated pDCs or lactate+ARC treated pDCs for 5 days before being intracellularly stained and acquired on a flow cytometer. *n* = 6 from 2 independent experiments and one-tailed Wilcoxon matched-pairs signed rank test was done. **(C)** Cell culture supernatants from pDC-T cell co-cultures, where pDCs were pre-treated as indicated, were subjected to acetonitrile extraction followed by LC-MS/MS to quantify the concentration of tryptophan and kynurenine in them. *n* = 8 from 3 independent experiments and two-tailed Wilcoxon matched-pairs signed rank test was done. **(D)** The ratio of kynurenine: tryptophan in the culture supernatants was correlated with the percentage of FoxP3^+^ cells in the co-cultures by Spearman's correlation (*N* = 4 in duplicate) (^*^p < 0.05, ^**^p < 0.005).

A major pro-tumorigenic property of tumor infiltrating pDCs is their ability to induce regulatory T cells (Tregs) thus adding to the immunosuppressive conditions in the tumor microenvironment (19, 20). Hence we explored if lactate also influences this aspect of intratumoral functional reprogramming of pDCs. We co-cultured lactate treated or untreated pDCs with autologous CD45RA^+^CD4^+^ naïve T cells and measured the abundance of FoxP3^+^ CD4^+^ T cells by flow cytometry (Figure 4A). We found that lactate-treated pDCs induced a higher percentage of FoxP3^+^ T cells, compared to untreated pDCs and this increased induction was abrogated in the presence of MCT inhibitor ARC (Figure 4B), thus implicating lactate transported into pDC cytosol, in this phenomenon. To further validate the identity of these FoxP3^+^ T cell, we stained for CD25 (an established cell surface marker for Tregs) along with a different clone of anti-FoxP3 antibody and found that indeed the lactate-induced CD4^+^FoxP3^+^ cells are highly positive for CD25 (Supplementary Figures S6A,B).

In order to confirm the immunosuppressive nature of these T cells, we isolated them by flow-sorting, co-cultured them with dye-labeled autologous CD45RA^+^ CD4^+^ T cells on anti-CD3/anti-CD28 antibody-coated plates and assessed the degree of proliferation by dye dilution. We found that the Tregs induced by the lactate-treated pDCs were indeed capable of suppressing autologous naïve CD4^+^ T cell proliferation (Supplementary Figures S7A,B).

Previous studies had reported that ability of pDCs to expand regulatory T cells may be mechanistically linked to either increased expression of ICOSL on pDCs enabling an ICOSL-ICOS mediated interaction with CD4 T cells (19, 40), or increased tryptophan metabolism leading to excessive production of kynurenines that in turn induces FoxP3 induction in T cells (41). We found that lactate fails to induce upregulation of ICOSL on human pDCs (Supplementary Figures S7C,D). On the other hand, we found that pDCs, cultured in the presence of lactate for 18 h, showed significantly higher tryptophan catabolism and secretion of kynurenine into the cell culture supernatants (detected by liquid chromatography of the supernatants followed by tandem mass spectrometry) (Supplementary Figures S8, S9). On inhibition of MCT-mediated lactate import into pDCs by adding ARC in the culture, the effect of lactate on pDC tryptophan metabolism was abolished (Supplementary Figure S9). This indicated that lactate transported into the cytosol via MCT drive the increase in kynurenine production. Furthermore, supernatant from 5 day co-culture of T cells with lactate treated pDCs had higher kynurenine: tryptophan ratio as opposed to supernatant from co-culture with either untreated pDCs or lactate-treated pDCs in presence of ARC (Figure 4C). As further validation for the link between lactate-induced kynurenine production by pDCs and generation of FoxP3^+^ CD4^+^ T cells, we explored the correlation between kynurenine abundance in pDC-T cell co-culture supernatants and the frequency of FoxP3^+^ CD4^+^ T cell generated after 5 days. Of note here, the correlation between these two parameters had to be done from a single experiment with multiple donors (*N* = 4) as the kynurenine flux and Treg frequency in co-cultures varied from experiment to experiment. We found strong positive correlation between the kynurenine: tryptophan ratio in the pDC: T cell co-culture supernatants and the percentage of FoxP3^+^ cells induced (Figure 4D). We concluded that lactate import into pDC cytosol via MCTs modulates pDC metabolism in a way that enables pDCs to induce FoxP3^+^ CD4^+^ regulatory T cells.

### Intratumoral pDCs Show Enhanced Tryptophan Metabolism and Higher Capacity to Induce FoxP3^+^CD4^+^ T Regulatory Cells

**Figure 5:**
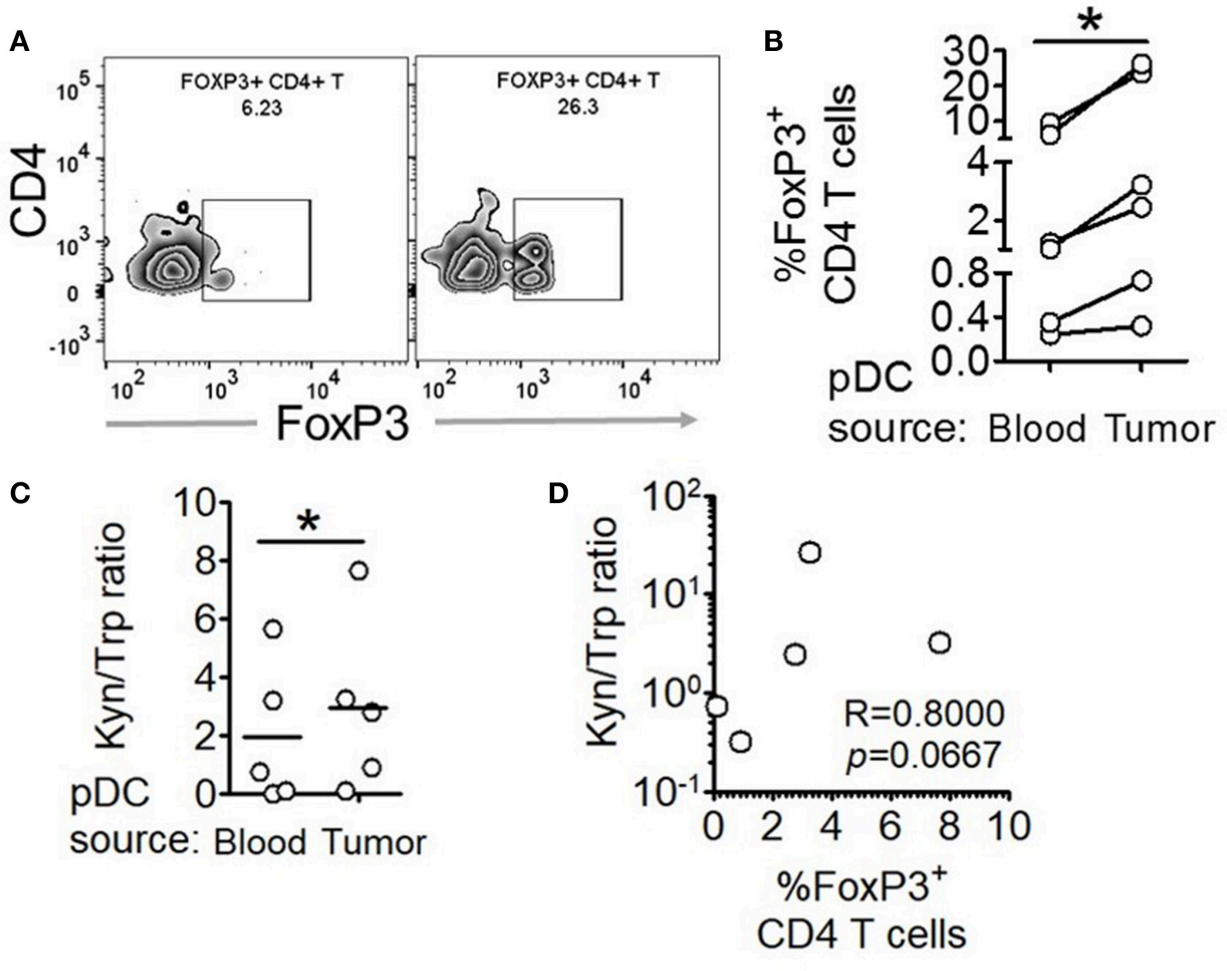
Intratumoral human pDCs expand FoxP3^+^ Tregs in breast cancer. **(A,B)** Blood and tumor tissue from patients with breast cancer was collected and processed to isolate pDCs, which were then co-cultured with autologous CD4^+^CD45RA^+^ T cells for 5 days, following which the cells were intracellularly stained for FoxP3 and subjected to flow cytometry. *n* = 6 from 6 independent experiments and two-tailed paired Student's *t*-test was done. **(C)** The culture supernatants from **(B)** were extracted and subjected to LC-MS/MS. Wilcoxon matched-pairs signed rank test was done. **(D)** The ratio of kynurenine: tryptophan in the culture supernatants was correlated with the percentage of FoxP3^+^ cells in the co-cultures by Spearman's correlation (^*^p < 0.05).

In order to validate our findings in *ex-vivo* tumor tissue samples, we isolated intratumoral and peripheral pDCs from patients with breast cancer, co-cultured them with autologous peripheral CD4^+^CD45RA^+^ T cells and measured the fraction of FoxP3^+^ cells by flow cytometric analysis (Figure 5A). Intratumoral pDCs (assumed to be exposed to higher concentrations of lactate) induced larger percentage of FoxP3^+^ T cells compared to peripheral pDCs (Figure 5B). Thus, this corroborates our *in-vitro* results by showing that tumor infiltrating pDCs derived from human breast tumors are capable of inducing more FoxP3^+^ CD4^+^ T cells, as well. Next, we explored whether aberrant tryptophan metabolism and excessive production of kynurenine by tumor infiltrating pDCs is responsible for this phenomenon in breast tumors as well. We found significantly enhanced kynurenine:tryptophan ratio in supernatants of co-cultures involving intratumoral pDCs compared to those involving peripheral pDCs from the same patients (Figure 5C). Moreover, there was positive correlation between the kynurenine: tryptophan ratio and the percentage of FoxP3^+^ cells induced (Figure 5D). Thus, tumor infiltrating pDCs from human breast cancer patients display enhanced tryptophan metabolism and are capable of inducing more FoxP3^+^ T cells.

### Lactate Abrogates IFNα Induction in Response to TLR9 Activation *in vivo*

**Figure 6:**
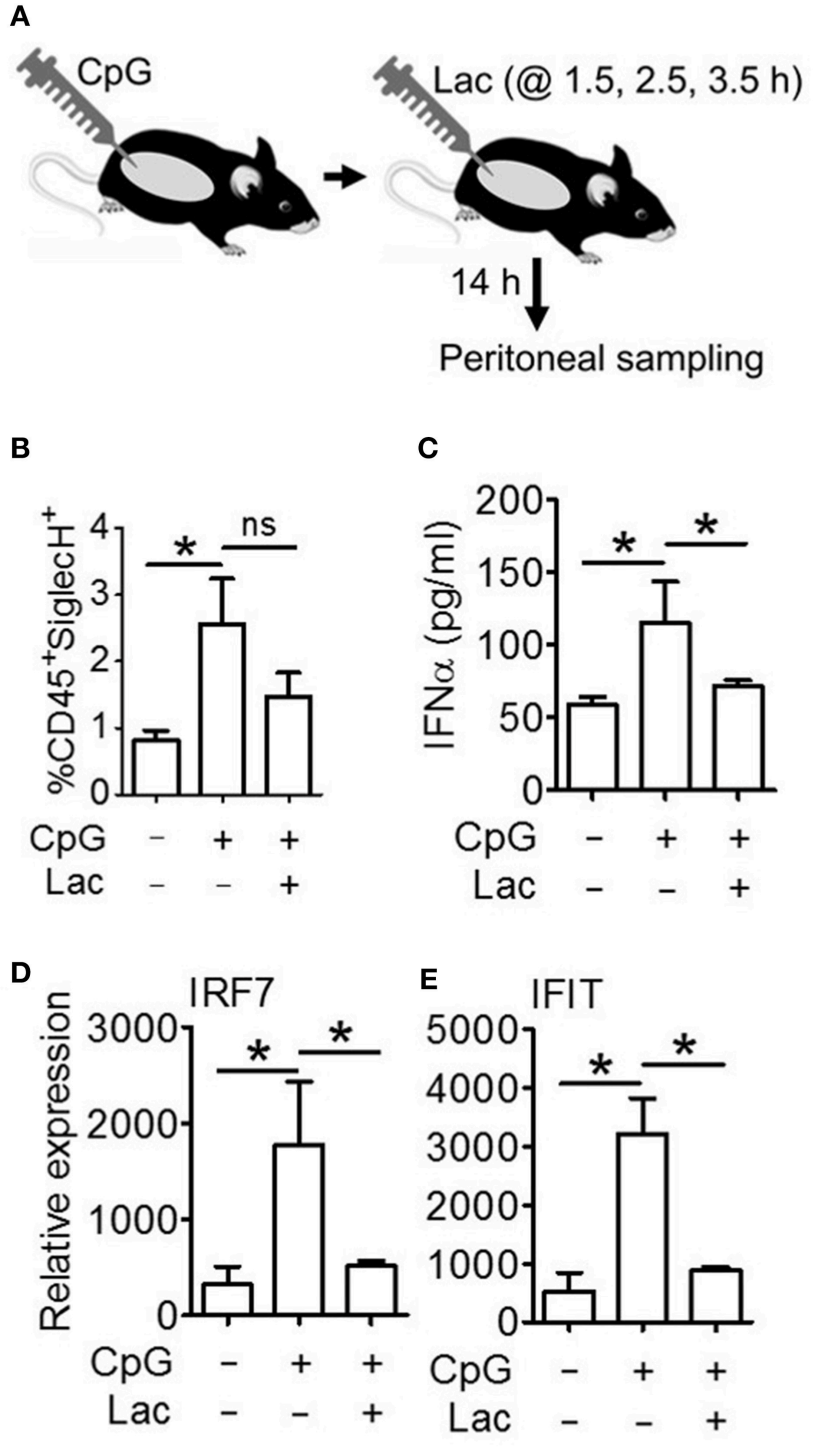
Lactate inhibits IFNα induction *in vivo*. **(A)** BALB/c mice were divided into 3 groups (Vehicle, CpG, and CpG+Lac) each having 3 mice The mice belonging to CpG and CpG+Lac groups were injected with CpG intraperitoneally followed by three doses of lactate at the indicated time points. Mice were sacrificed after 14 h, following which peritoneal wash with 1X PBS was collected and centrifuged to separate the liquid portion from the cellular portion which were then subjected to further downstream processing. **(B)** Peritoneal cells were stained with CD45 PerCP and SiglecH BV421 before flow cytometric acquisition to determine the percentage of infiltrating pDCs. **(C)** Peritoneal wash was subjected to mouse IFNα ELISA to determine its concentration in peritoneal fluid. **(D,E)** Gene expression studies were done on the isolated peritoneal cells to measure the expression of ISGs- IRF7 **(D)** and IFIT **(E)** relative to the expression of the housekeeping gene 18S. *n* = 3 from 1 independent experiment and Mann-Whitney test was done. Data is represented as Mean ± SEM (^*^p < 0.05 and ns, non-significant).

Next, in order to decipher whether these oncometabolite-mediated immune evasion pathway are operative *in vivo* as well, we performed an *in vivo* efficacy assay wherein we injected CpG stimulated BALB/c mice with 0.5 g/kg lactate (Figure 6A), and found significantly lesser IFNα accumulation in peritoneal fluid (Figure 6C) as well as lesser expression of Interferon Signature Genes (IRF7 and IFIT) in peritoneal cells (Figures 6D,E) compared to mice which had been stimulated with CpG alone—though both groups had comparable pDC infiltration in peritoneum (Figure 6B). Thus, pathways responsible for inhibition of IFNα production pathways by lactate is operative *in vivo* as well.

### Intratumoral Injection of Gallein and ARC Cause Significant Reduction in Tumor Burden

**Figure 7:**
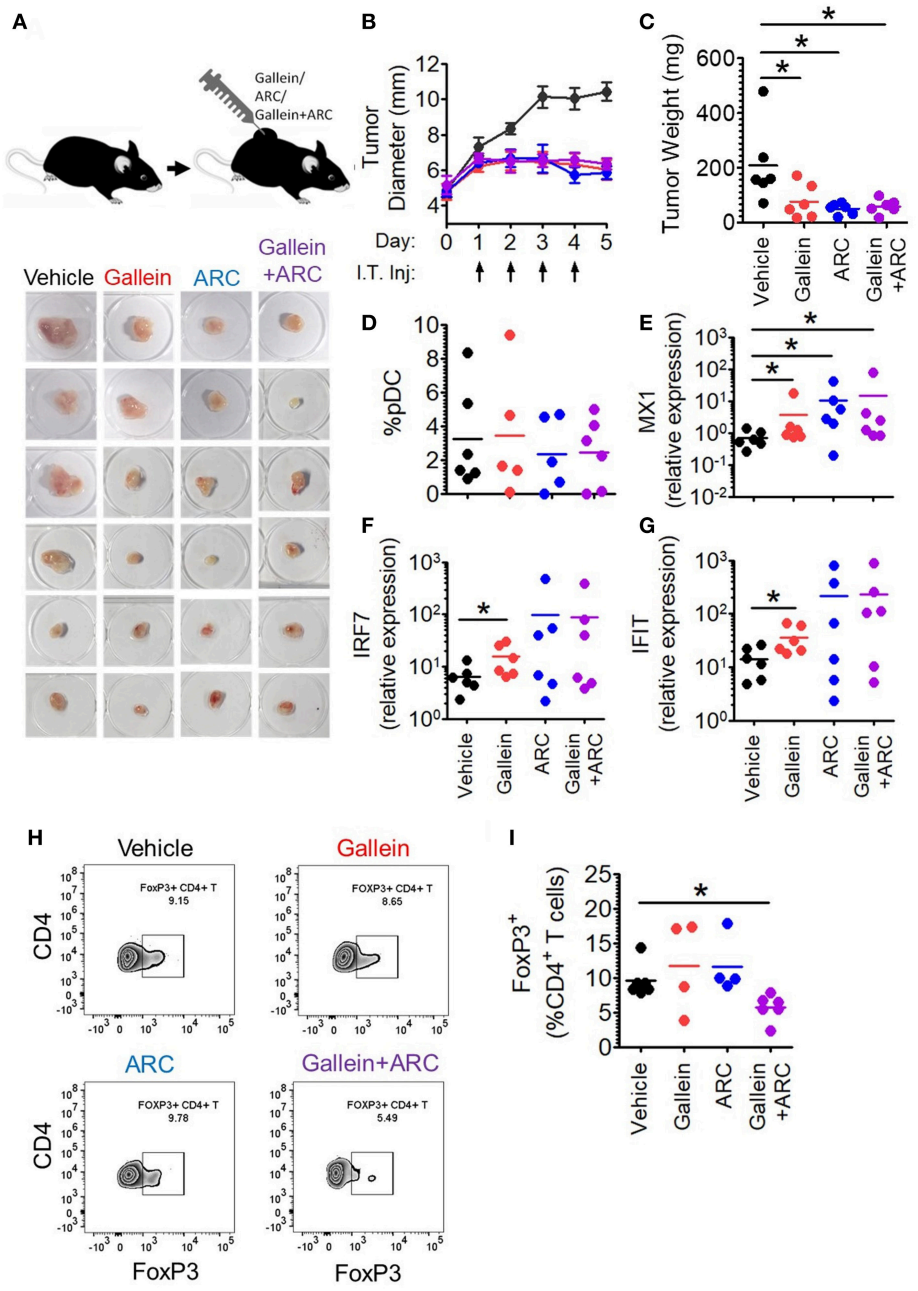
Intratumoral gallein and ARC recovers type I IFN induction and reduces Treg expansion in syngeneic breast cancer model in mice. **(A)** 4T1 tumor bearing BALB/c mice were divided into 4 groups each having 6 mice. Daily intratumoral injection (5 μl) of either PBS, 160 ng gallein alone, 18.5 ng ARC alone or ARC in combination with gallein was done for each mouse for 4 consecutive days. **(B)** The diameter of the tumors was recorded each day with a vernier caliper and compared between the different groups. Data is from 3 independent experiments and represented as Mean ± SEM. **(C)** On the 5th day mice were sacrificed and tumors excised, weighed and imaged. Data is from 3 independent experiments. One-tailed unpaired *t*-test was done. **(D)** Tumors from each group of mice were digested and the cellular suspension was stained with anti-mouse CD45 PerCP, Siglec-H BV421, and PDC-TREM PE antibodies before being subjected to flow cytometry to determine the percentage of pDCs. Data is from 3 independent experiments and one-tailed unpaired *t*-test was done. **(E–G)** Real-time PCR was done to measure the expression levels of 3 ISGs-MX1 **(E)**, IRF7 **(F)**, and IFIT **(G)** in tumor tissue. Mann-Whitney test was done (*n* = 6 from 3 independent experiments). **(H,I)** Staining with anti-mouse CD45 PerCP, TCR-β APC-Cy7, CD4 APC, and FoxP3 V450 (intracellular staining) was done to measure the percentage of intratumoral FoxP3^+^ CD4^+^ T cells by flow cytometry. Data is from 3 independent experiments and two-tailed unpaired *t*-test was done (^*^p < 0.05).

Next, in order to further explore the *in vivo* efficacy of inhibition of lactate signaling pathway in mouse preclinical breast cancer model, we developed 4T1 breast tumor model in BALB/c mice. We let the tumors grow to an average diameter of 6.5 mm before injecting them daily with the given doses of gallein (the inhibitor of GPR81 mediated pathway) and/or ARC (the MCT inhibitor) for 4 consecutive days (Figure 7A). Gallein alone, ARC alone as well as the two in combination, caused sharp retardation in tumor growth (Figure 7B). This was further validated by the weights of the excised tumors which were significantly less in the inhibitor treated groups compared to the vehicle treated group (Figure 7C). Thus, inhibition of lactate signaling pathways in 4T1 breast tumor containing BALB/c mice caused significant reduction in tumor load indicating both GPR81 and MCTs as potential targets for future chemotherapeutic drugs needing further investigation.

### Inhibition of Pathways Involved in Lactate Signaling Enhances Intratumoral Interferon Signature and Reduces Expansion of FoxP3^+^ Cells

It is well-known that lactate exerts both cancer cell intrinsic and extrinsic effects (27–31). Hence, inhibitors of lactate signaling pathways must also have both kinds of effects which together might be responsible for the reduction in tumor burden. But, our interest lay in the effect of these inhibitors, *in vivo*, on the intratumoral functional reprogramming of pDCs and expansion of regulatory T cells, as a proof of principle for our *in vitro* studies. Toward this, we compared the percentage of tumor infiltrating pDCs as well as the expression of Interferon Signature Genes (ISGs-representative of the extent of interferon signaling) in the tumor tissue upon injection of gallein alone, ARC alone or the two in combination, with vehicle treated control group mice. There was appreciable pDC infiltration in tumor tissues with no significant difference in terms of extent recruitment among the four group of mice (Figure 7D). Intratumoral injection of gallein caused a significant increase in the expression of ISGs such as MX1, IRF7, and IFIT, thus supporting our *in-vitro* data. This recovery of intratumoral IFN induction was more variable in response to concomitant MCT inhibition (Figures 7E–G). This can be explained by the fact that TLR stimulation in pDCs should lead to accumulation of lactate in the cytosol due to the glycolytic switch in cellular respiration and that should also have negative feed-back on glycolysis as well as type I IFN induction. As MCTs can mediate both passive extracellular export of lactate and intracellular import, inhibiting them in the presence of low levels or absence of extracellular lactate should actually inhibit type I IFN induction by helping in endogenous lactate accumulation. Accordingly, in the absence of extracellular lactate, ARC dampened type I IFN induction by human pDCs as well in response to TLR9 ligand *in vitro* (Supplementary Figure S10A). On the other hand gallein had no effect on IFNα induction by human pDCs in the absence of exogenous lactate (Supplementary Figure S10B), as expected, since it is known to inhibit the cell surface receptor mediated arm of lactate signaling and should not affect endogenous lactate-mediated signaling. Thus, it was evident that in 4T1 syngeneic breast tumor model in mice too, lactate mediated immunosuppression of intratumoral type I IFN induction do occur and inhibition of the two major arms of lactate signaling causes increased interferon signaling. When we compared the percentage of FoxP3^+^ CD4^+^ T cells between the control and the treated groups, we could see significant decrease in the percentage of FoxP3^+^ CD4^+^ T cells when both gallein and ARC were injected (Figures 7H,I). Thus, we found that the lactate-induced functional reprogramming of human pDCs was also operative in this syngeneic mouse tumor model *in vivo* and interference with the identified pathways affected tumor growth.

**Figure 8:**
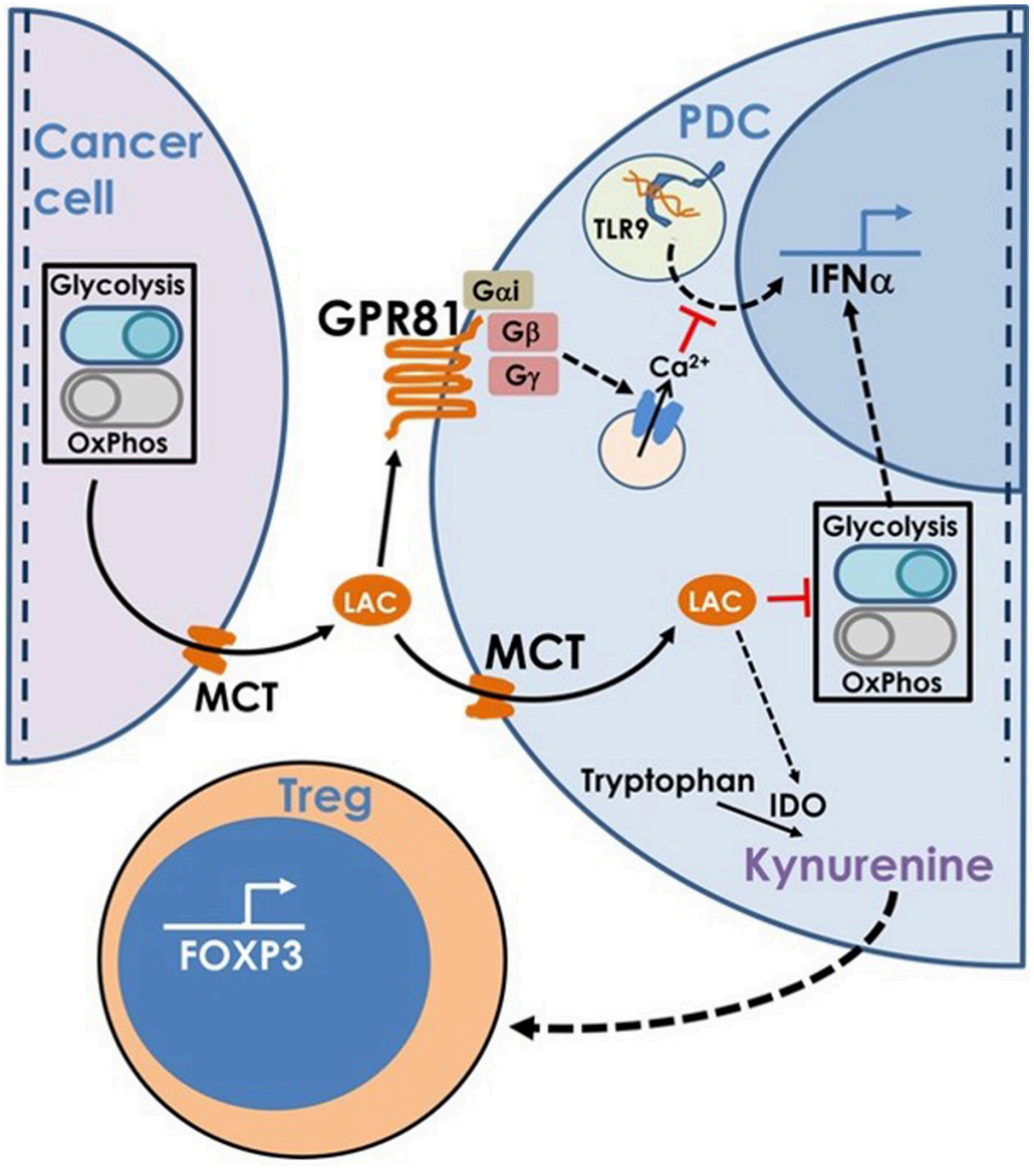
This model is based on our findings that the excess lactate secreted by cancer cells as a result of characteristic adaptations in their metabolic profile, leads to suppression of type I IFN induction in pDCs, via two distinct cellular mechanisms. Binding of lactate to GPR81 receptor on pDC surface leads to intracellular calcium mobilization which inhibits IFNα production as well as influences the glycolytic switch. Import of lactate through monocarboxylate transporters and its subsequent accumulation in the pDC cytosol also inhibits the CpGA induced glycolytic switch essential for IFNα production. Apart from this, lactate transported into pDC cytosol also enhances tryptophan catabolism leading to excessive production of kynurenine which in turn leads to Treg expansion, thus adding to the overall immunosuppression in the tumor microenvironment.

Hence, to summarize, lactate produced by cancer cells inhibits type I IFN induction by pDCs by binding to GPR81 on pDC surface or via transport into pDC cytosol via MCT. In addition, once transported into pDC cytosol, lactate enhances tryptophan catabolism and kynurenine production by pDCs thus enhancing Treg expansion (Figure 8).

## Discussion

Tumorigenesis does trigger anti-tumor immune activation, and the interaction evolves through a phase of relative equilibrium before finally getting subverted by the proliferating tumor cells (1). Gradual development of an immuno-suppressive milieu in the micro-environment in addition to immunoediting of the cancer cells contributes to cancer growth and eventual immune escape. Metabolic adaptation and a characteristic cellular respiration with glycolytic dominance play a crucial role in enabling cancer cells to balance between energy expenditure and macromolecular biosynthesis to support high rates of proliferation (21). Lactic acid is the major metabolite released by the cancer cells as a consequence of these metabolic adaptations (26). Several studies now show that this metabolite can actually link the pro-growth metabolic adaptations to immune-suppression in cancer (27), with evidence for suppressive effects against myeloid cells (28, 30), NK cells and cytotoxic T cells (29). Here we show that lactate also attenuates IFNα induction in response to TLR ligands by pDCs, the most important cellular producers, thus evading a critical component of anti-tumor immunity. Lactate also enhances the ability of pDCs to induce regulatory T cells which are well-known components of an immunosuppressive milieu in the TME of multiple types of solid tumors associated with a poor prognosis.

PDC infiltration into the tumor bed has been associated with poor prognosis in breast cancer and pDC-driven expansion of regulatory T cells has been implicated in this immunosuppression (19, 20). But how do the tumors evade intra-tumoral type I IFN induction by pDCs, despite being rich sources of TLR9 and TLR7 ligands due to high cell turn over and cell death, is far from being clear. Suppressive role of tumor-derived TNFα and TGFβ on pDCs have been implicated in a previous study (20). We found here that cancer cell-derived lactate, through the cell surface lactate receptor GPR81 as well as via intracellular transport through MCTs, can potently inhibit TLR signaling in pDCs as depicted in the model. We presume that these two mechanisms either co-operate for this immune-evasion or one may become more dominant based on specific microenvironmental context (Figure 8). The GPR81-mediated inhibition was dependent upon Gβγ-dependent cytosolic free Ca^2+^ mobilization, without any effect of the Gαi signaling. This was not unexpected, as cAMP accumulation itself has previously been shown to inhibit type I IFN induction in intra-tumoral pDCs (42). On the other hand MCT-mediated cytosolic import of lactate interferes with the similar metabolic adaptation required by activated pDCs rendering them dysfunctional.

We also explored the effect of lactate on the ability of pDCs to induce Tregs since the presence of Tregs in the tumor microenvironment is strongly associated with poor prognosis. It has been proposed earlier that ICOSL overexpression on intratumoral pDCs enable them to expand Tregs through ICOSL-ICOS interaction (19, 40). But we found lactate does not drive ICOSL overexpression in human pDCs. Previous reports have also implicated tryptophan catabolism leading to the production of kynurenine as the causal factor for pDC mediated Treg induction (41) and kynurenine is known to induce Tregs via interaction with the aryl hydrocarbon receptor (43). We found that lactate enhances tryptophan catabolism and the production of kynurenine by pDCs which is strongly correlated with their increased ability to induce FoxP3^+^ Tregs compared to untreated pDCs. Moreover, intratumoral pDCs showed the same characteristics of elevated tryptophan catabolism as well as FoxP3 induction, unlike peripheral pDCs of patients with breast cancer. Moreover, intraperitoneal injection of lactate abrogated IFNα induction thus confirming its inhibitory effect on IFNα production *in vivo*. Finally, intratumoral injection of a combination of lactate signaling pathway inhibitors gallein and ARC in 4T1 tumor mouse models, led to significant reduction in the percentage of intratumoral FoxP3^+^ CD4^+^ T cells, thus confirming the role of lactate in enhancing Treg induction.

We think that this oncometabolic-driven reprogramming of pDC function in a tumor is a critical immune-evasive mechanism, which in turn contributes to dysregulation of other innate and adaptive mechanisms of anti-tumor immunity. A critical role of type I IFNs in the regulation of NK cells is well-documented (44). Anti-tumor effects of NK cells have been shown to be critically regulated by type I IFNs (45). A potential role of type I IFNs in driving pro-inflammatory polarization of tissue-recruited macrophages has also been reported recently (16). Thus, cancer cell-derived lactate-driven attenuation of pDC function can actually result in multiple immune dysfunctions, contributing greatly to the immune escape of tumors. Same is true for intratumoral Treg expansion, for which intratumoral functional reprogramming of pDCs has been shown to be involved in previous reports as well (19, 20, 40). Administration of immune checkpoint blocking agents or TLR-agonists are promising immunotherapeutic strategies against cancer, but to circumvent issues with variability and multiplicity of immunosuppressive mechanisms more efficacious combinatorial strategies targeting microenvironmental factors are essential (46). The mechanistic insights into this lactate-driven attenuation of two critical components of anti-tumor immune response creates possibility of developing new generation therapies, targeting key events in this immuno-evasion pathway (e.g., the GPR81 receptor or the MCT transporters), that can further potentiate the usual anti-cancer immunotherapeutic strategies.

Thus, we could identify a hitherto unknown link between intra-tumoral abundance of the oncometabolite lactate, resulting from metabolic adaptation in cancer cells, and the pro-tumor tolerogenic reprogramming of plasmacytoid dendritic cells. We also could identify non-redundant role of the cell surface lactate receptor GPR81 and the monocarboxylate transporters in mediating the effects of lactate on pDCs, in terms of both IFNα induction as well as ability to expand regulatory T cells, revealing new therapeutic targets for potentiating anti-cancer immune responses. We could validate our model by looking into tumor recruited pDCs from patients with breast cancer, as well as in a syngeneic model of breast cancer in mice.

## Data Availability

All datasets generated for this study are included in the manuscript and/or the Supplementary Files.

## Ethics Statement

All experiments with patient samples were done as per recommendations of the Institutional Review Boards of Institute of Postgraduate Medical Education & Research, Kolkata, India as well as CSIR-Indian Institute of Chemical Biology (IICB), Kolkata, India, and after obtaining informed consent from the donors. The human study was conducted on de-identified samples. All animal experiments were done on approval of the Institutional Animal Ethics Committee of CSIR-IICB.

## Author Contributions

DG conceived and designed the study. DR did most of the experiments. RB, AG, and OR participated in the experiments with patient samples. CL, OR, and PB helped with the pDC functional assays and gene expression studies. SC helped in designing the *in vivo* experiments. BS, AG, JS, and RD'R helped with the *in vivo* experiments. SP helped with the LC-MS/MS studies. AD and DS recruited the patients. DG and DR wrote the manuscript.

### Conflict of Interest Statement

The authors declare that the research was conducted in the absence of any commercial or financial relationships that could be construed as a potential conflict of interest.
