# Supplementary material for: Lactate Induces Pro-tumor Reprogramming in Intratumoral Plasmacytoid Dendritic Cells
Source: Front Immunol. 2019 Aug 7;10:1878. doi: 10.3389/fimmu.2019.01878 (PMC6692712; doi:10.3389/fimmu.2019.01878)
Supplement: Supplementary file 1 [file Data_Sheet_1.PDF]

# Supplemental materials

**For**

# ‘Lactate induces pro-tumor reprogramming in intratumoral plasmacytoid dendritic cells’

**by**

**Deblina Raychaudhuri et al.**

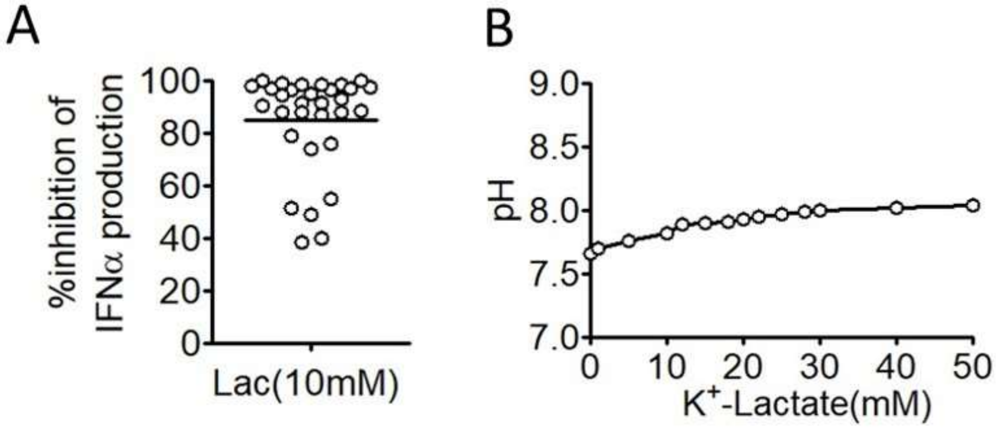


**Supplementary Figure S1**

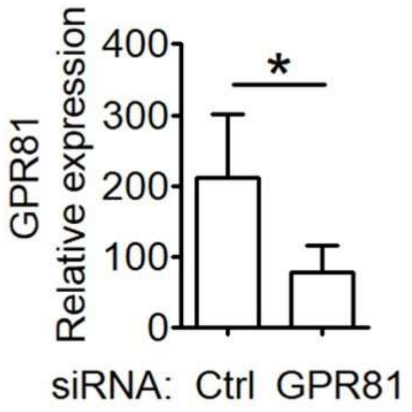


**Supplementary Figure S2**

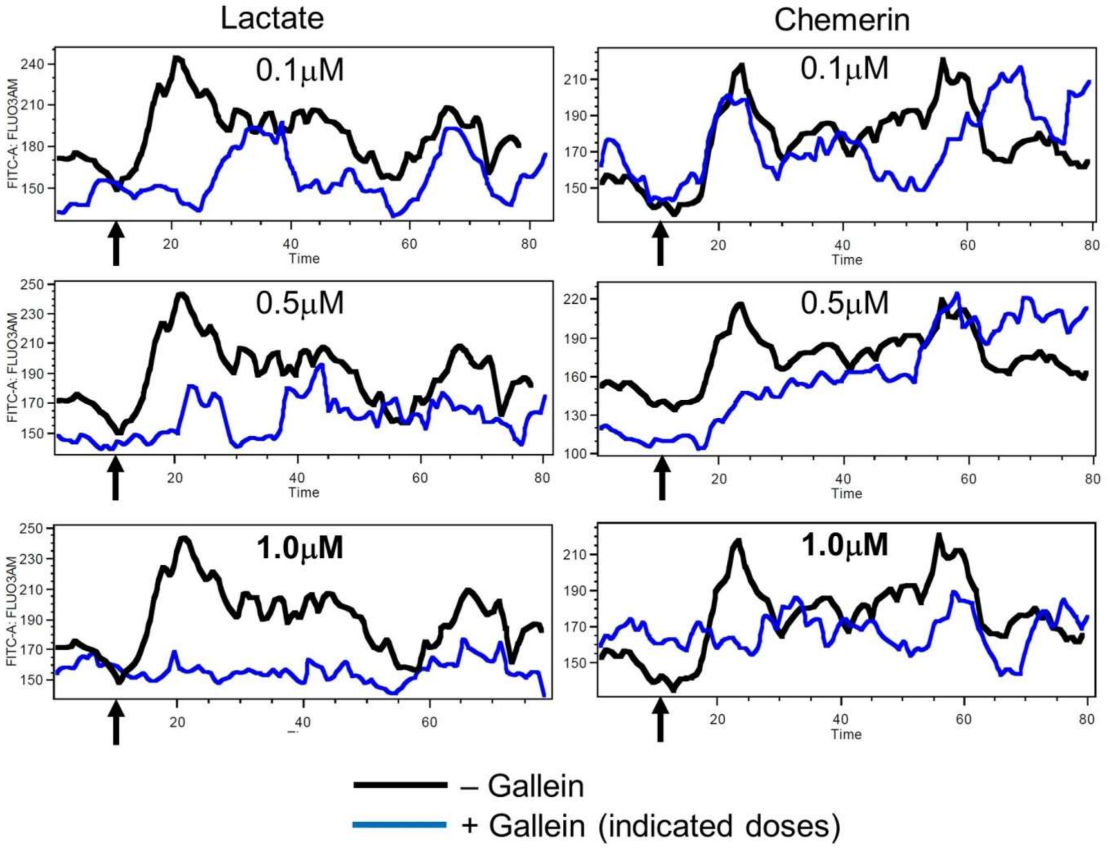


**Supplementary Figure S3**

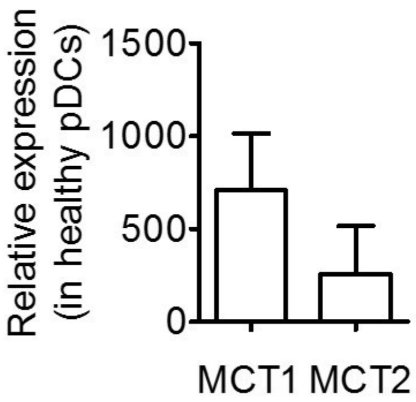


**Supplementary Figure S4**

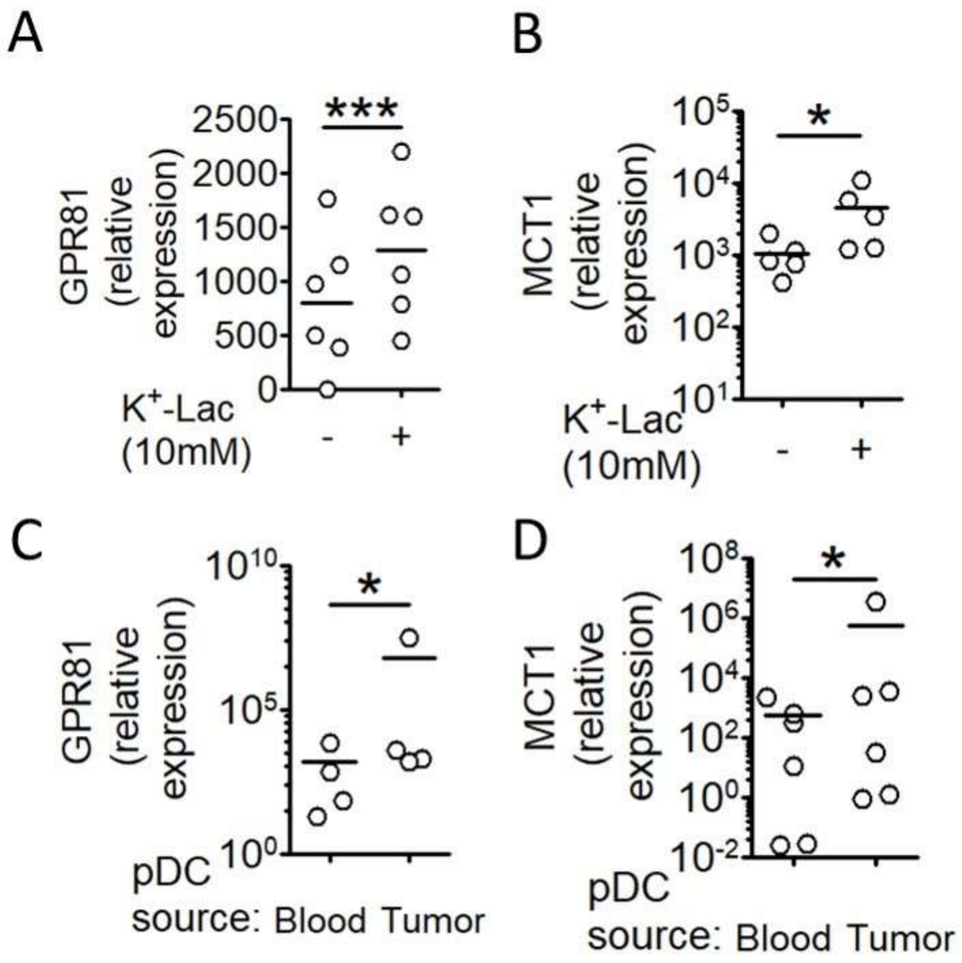


# Supplementary Figure S5

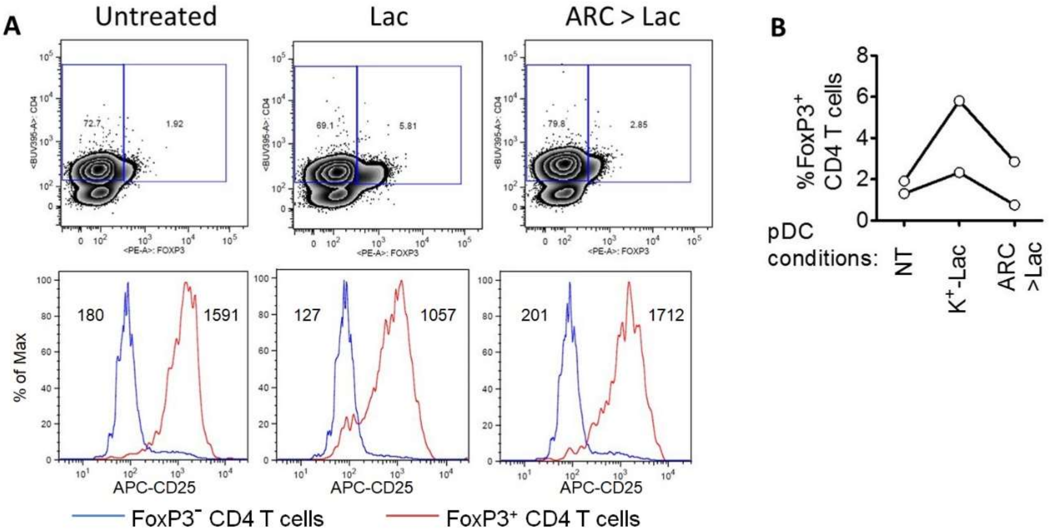


**Supplementary Figure S6**

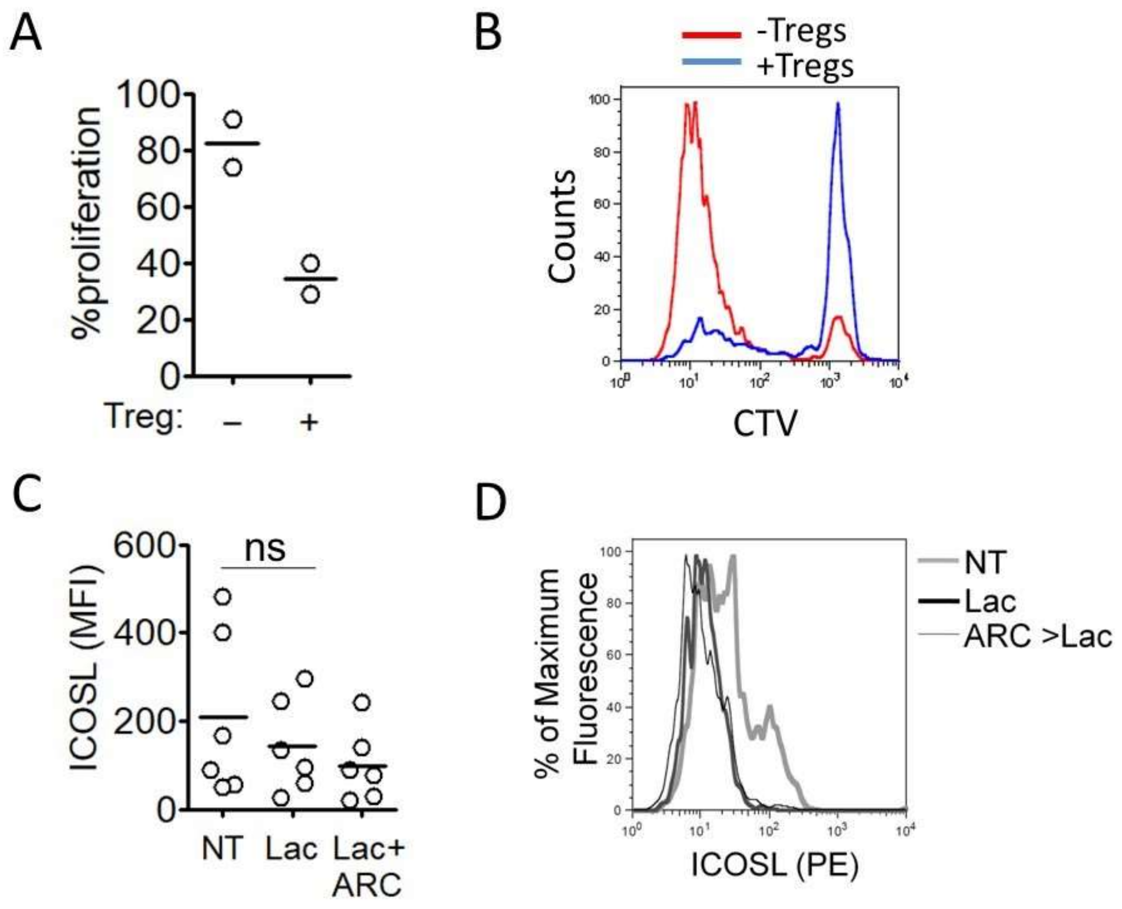


**Supplementary Figure S7**

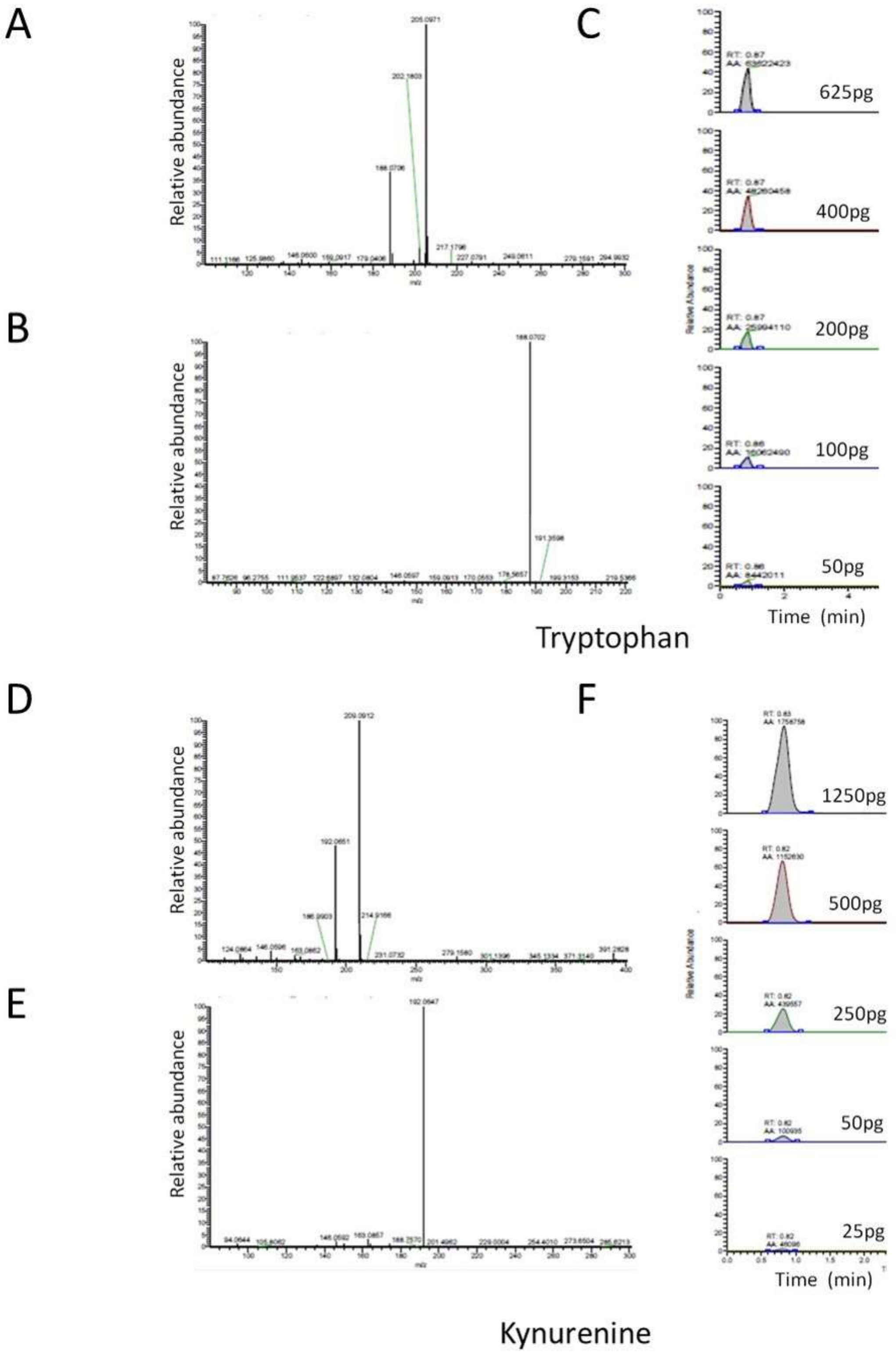


**Supplementary Figure S8**

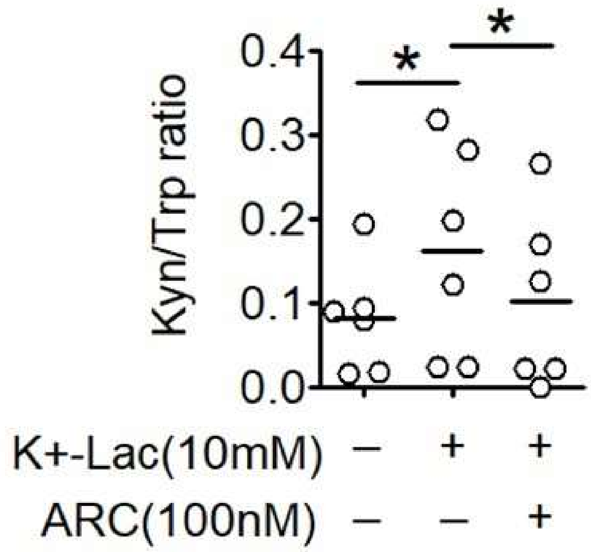


**Supplementary Figure S9**

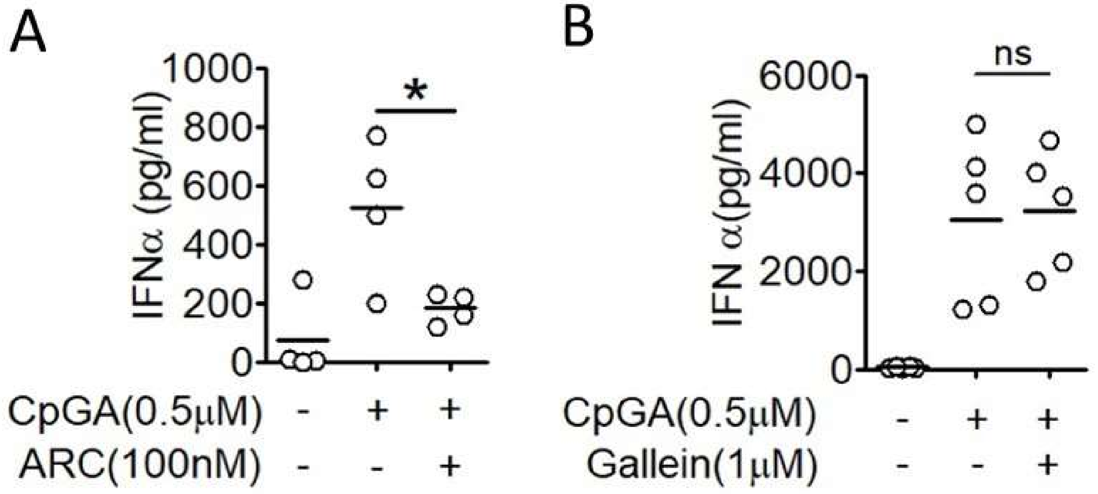


**Supplementary Figure S10**

**Supplemental Table 1.** Primers used in real time PCR experiments

| Gene | Primer sequence |
|---|---|
| h18S | F: 5'-GTAACCCGTTGAACCCCATT-3'<br>R: 5'-CCATCCAATCGGTAGTAGCG-3' |
| hGPR81 | F: 5'-TGGGCGCACTAGGCAATGGG-3'<br>R: 5'-GTCCGCAGCCACCACCGTAA-3' |
| hMCT1 | F: 5'-TGCCACCACCAGCGAAGTGT-3'<br>R: 5'-AGGCAAGCCCAAGACCTCCA-3' |
| hMCT2 | F: 5'-TGCGCCAGAGACCAGATAAAGATCA-3'<br>R: 5'-CTCCAACCACAATCCAACCCCAT-3' |
| m18S | F: 5'-GTTGGTTTTCGGAACTGAGG-3'<br>R: 5'-TCGTTTATGGTCGGAACTACG-3' |
| mIRF7 | F: 5'-TCAGAAGCAGCTGCACTACACAGA-3'<br>R: 5'-TACCTCCCAGTACACCTTGCACTT-3' |
| mMX1 | F: 5'-TTCAAGGATCACTCATACTTCAGC-3'<br>R: 5'-GGGAGGTGAGCTCCTCAGT-3' |
| mIFIT1 | F: 5'-AGGCTGGAGTGTGCTGAGAT-3'<br>R: 5'-TCTGGATTTAACCGGACAGC-3' |

**Supplemental Table 2.** Patient details

| | | |
|---|---|---|
| Number | 10 | |
| Age | 45.8 ± 7.786 years (Mean ± standard deviation) | |
| Antigen status | ER+ PR+ HER2+ | 2 |
| | ER+ PR+ HER2- | 2 |
| | ER- PR- HER2- | 3 |
| | ER- PR- HER2+ | 2 |
| | ER- PR+ HER2- | 1 |

**FIGURE LEGEND FOR SUPPLEMENTARY FIGURES**

**Fig.S1) Lactate inhibits type IFNα induction by pDCs. S1A)** The % inhibition of IFNα in culture supernatants of pDCs caused by treatment with 10mM K+-lactate before CpGA stimulation.(n=31). **S1B)** Various doses of K+-Lac were added to complete complete RPMI media and the pH was measured in a calibrated pH meter.

**Fig.S2) Knockdown efficiency of GPR81 in human pDCs..** The expression levels of GPR81 transcript relative to that of 18S (housekeeping gene) was measured in pDCs nucleofected with either control siRNA or GPR81 siRNA after 16 hours of culture.(n=8). Paired Student's *t*-test was performed. Normalized expression data (mean ± SEM) are presented. $^{*}p<0.05$.

**Fig.S3) Dose response of gallein mediated abrogation of GPCR-induced calcium mobilisation in pDCs.** Fluo 3-AM stained primary pDCs were pre-incubated with indicated doses of gallein before acquisition in a flow cytometer to measure calcium mobilisation before and after addition of 10ng/ml chemerin or 10mM lactate as indicated. Graph is representative of 3 independent experiments.

**Fig.S4) Human pDCs express both MCT1 and MCT2.** Expression data of the monocarboxylate transporters MCT1 and MCT2 on human pDCs relative to the expression of 18S housekeeping gene. Data (mean ± SEM) are presented.

**Fig.S5) Exposure to lactate enhances the expression of both GPR81 and MCT1 in human pDCs. S5A,B,C,D)** pDCs isolated from tumour tissue and peripheral blood of breast cancer patients(C,D), as well as peripheral pDCs from healthy donors treated as indicated (A,B), were subjected to gene expression studies and the relative expression levels of 2 genes-GPR81 (A,C) and MCT1 (B,D) normalised to the internal control-18S gene expression, were checked by Real-time PCR. Paired Student's *t*-test was done. . $^{*}p<0.05$, $^{***}p<0.005$.

**Fig.S6) Lactate induces FoxP3$^{+}$ CD4$^{+}$ T cells with high CD25 expression.** Autologous CD45RA$^{+}$CD4$^{+}$ T cells were co-cultured with untreated pDCs, K+-Lac treated pDCs or lactate+ARC treated pDCs for 5 days before being stained and acquired on a flow cytometer (n=2 from one independent experiment). **S6A)** Flow cytometric assessment of FoxP3+ CD4+ T cells in culture with pDCs treated with indicated conditions (top panel) and comparison of CD25 expression on FoxP3- and FoxP3+ T cells (bottom panel: blue line FoxP3-, red line FoxP3+, respective MFIs are indicted in the histogram). **S6B)** Comparison of FoxP3+ CD4+ T cells in different conditions.

**Fig.S7) Lactate-driven pDCs generate Tregs independent of ICOSL upregulation. S7A,B)** CD4$^{+}$CD25$^{high}$CD127$^{low}$ cells were sorted from pDC co-cultures using a cell sorter and once again co-cultured with freshly isolated CTV stained naive T cells from autologous donors. After 5 days, the dilution of CTV was recorded in a flow cytometer as a measure of the degree of proliferation. One representative histogram for the flow cytometric proliferation assay is shown in S7B. **S7C,D)** Primary human pDCs, treated as indicated overnight in culture, were stained and acquired in a flow cytometer to measure the expression of ICOSL on the cell surface. Paired Student's *t*-test was done. One representative histogram is shown in S7D.

**Fig. S8) Mass spectrometry for the detection of tryptophan and kynurenine. S8A,B)** The major peak of tryptophan obtained after LC-MS having a m/z of 205.0971 (A) and after LC-MS/MS having a m/z of 188.0702 (B). **S8C)** Intensity of the peak of tryptophan obtained at indicated amounts of tryptophan.

**S8D,E)** The major peak of kynurenine obtained after LC-MS having a m/z of 209.0912 (D) and after LC-MS/MS having a m/z of 192.0647 (E). S8F) Intensity of the peak of tryptophan obtained at indicated amounts of kynurenine.

**Fig. S9) Lactate transported through MCTs enhances tryptophan catabolism in pDCs.** Cell culture supernatants from pDCs cultured in the presence of indicated treatments were subjected to acetonitrile extraction followed by LC-MS/MS to quantify the concentration of tryptophan and L-kynurenine in them. Wilcoxon matched-pairs signed rank test was done. $*p<0.05$.

**Fig. S10) Differential effect of ARC and gallein on IFNα induction by pDCs. S10A,B)** IFNα ELISA was done on supernatants from pDC cultures containing indicated concentrations of ARC (A) or gallein (B) and stimulated with CpGA. Paired Student's *t*-test was done. $*p<0.05$, *ns*=nonsignificant.
